# Algal Functional Annotation Tool: a web-based analysis suite to functionally interpret large gene lists using integrated annotation and expression data

**DOI:** 10.1186/1471-2105-12-282

**Published:** 2011-07-12

**Authors:** David Lopez, David Casero, Shawn J Cokus, Sabeeha S Merchant, Matteo Pellegrini

**Affiliations:** 1 Department of Molecular, Cell, and Developmental Biology, University of California, Los Angeles, CA, USA; 2 Department of Chemistry and Biochemistry, University of California, Los Angeles, CA, USA; 3 Institute of Genomics and Proteomics, University of California, Los Angeles, CA, USA

## Abstract

**Background:**

Progress in genome sequencing is proceeding at an exponential pace, and several new algal genomes are becoming available every year. One of the challenges facing the community is the association of protein sequences encoded in the genomes with biological function. While most genome assembly projects generate annotations for predicted protein sequences, they are usually limited and integrate functional terms from a limited number of databases. Another challenge is the use of annotations to interpret large lists of 'interesting' genes generated by genome-scale datasets. Previously, these gene lists had to be analyzed across several independent biological databases, often on a gene-by-gene basis. In contrast, several annotation databases, such as DAVID, integrate data from multiple functional databases and reveal underlying biological themes of large gene lists. While several such databases have been constructed for animals, none is currently available for the study of algae. Due to renewed interest in algae as potential sources of biofuels and the emergence of multiple algal genome sequences, a significant need has arisen for such a database to process the growing compendiums of algal genomic data.

**Description:**

The Algal Functional Annotation Tool is a web-based comprehensive analysis suite integrating annotation data from several pathway, ontology, and protein family databases. The current version provides annotation for the model alga *Chlamydomonas reinhardtii*, and in the future will include additional genomes. The site allows users to interpret large gene lists by identifying associated functional terms, and their enrichment. Additionally, expression data for several experimental conditions were compiled and analyzed to provide an expression-based enrichment search. A tool to search for functionally-related genes based on gene expression across these conditions is also provided. Other features include dynamic visualization of genes on KEGG pathway maps and batch gene identifier conversion.

**Conclusions:**

The Algal Functional Annotation Tool aims to provide an integrated data-mining environment for algal genomics by combining data from multiple annotation databases into a centralized tool. This site is designed to expedite the process of functional annotation and the interpretation of gene lists, such as those derived from high-throughput RNA-seq experiments. The tool is publicly available at http://pathways.mcdb.ucla.edu.

## Background

Next-generation sequencers are revolutionizing our ability to sequence the genomes of new algae efficiently and in a cost effective manner. Several assembly tools have been developed that take short read data and assemble it into large continuous fragments of DNA. Gene prediction tools are also available which identify coding structures within these fragments. The resulting transcripts can then be analyzed to generate predicted protein sequences. The function of these protein sequences are subsequently determined by searching for close homologs in protein databases and transferring the annotation between the two proteins. While some versions of the previously described data processing pipeline have become commonplace in genome projects, the resulting functional annotation is typically fairly minimal and includes only limited biological pathway information and protein structure annotation. In contrast, the integration of a variety of pathway, function and protein databases allows for the generation of much richer and more valuable annotations for each protein.

A second challenge is the use of these protein-level annotations to interpret the output of genome-scale profiling experiments. High-throughput genomic techniques, such as RNA-seq experiments, produce measurements of large numbers of genes relevant to the biological processes being studied. In order to interpret the biological relevance of these gene lists, which commonly range in size from hundreds to thousands of genes, the members must be functionally classified into biological pathways and cellular mechanisms. Traditionally, the genes within these lists are examined using independent annotation databases to assign functions and pathways. Several of these annotation databases, such as the Kyoto Encyclopedia of Genes and Genomes (KEGG) [1], MetaCyc [2], and Pfam [3], include a rich set of functional data useful for these purposes.

However, presently researchers must explore these different knowledge bases separately, which requires a substantial amount of time and effort. Furthermore, without systematic integration of annotation data, it may be difficult to arrive at a cohesive biological picture. In addition, many of these annotation databases were designed to accommodate a single gene search, a methodology not optimal for functionally interpreting the large lists of genes derived from high-throughput genomic techniques. Thus, while modern genomic experiments generate data for many genes in parallel, their output must often still be analyzed on a gene-by-gene basis across different databases. This fragmented analysis approach presents a significant bottleneck in the pipeline of biological discovery.

One approach to solving this problem is integrating information from multiple annotation databases and providing access to the combined biological data from a single comprehensive portal that is equipped with the proper statistical foundations to effectively analyze large gene lists. For example, the DAVID database integrates information from several pathway, ontology, and protein family databases [4]. Similarly, Ingenuity Pathway Analysis (IPA) provides an integrated knowledge base derived from published literature for the human genome [5]. The integrated functional information and annotation terms are then assigned to lists of genes and for some analyses, enrichment tests are performed to determine which biological terms are overrepresented within the group of genes. By combining the information found in a number of knowledge bases and performing the analysis of lists of genes, these tools permit the efficient processing of high-throughput genomic experiments and thus expedite the process of biological discovery. However, most of these integrated databases have been developed for the analysis of well-annotated and thoroughly studied organisms, and are lacking for many newly genome-enabled organisms.

One large group of organisms for which integrated functional databases are lacking are the algae. The algae constitute a branch in the plant kingdom, although they form a polyphyletic group as they do not include all the descendants of their last common ancestor. As many as 10 algal genomes have been sequenced, including those of a red alga and several chlorophyte algae, with several more in the pipeline [6-11]. Algal genomic studies have provided insights into photosymbiosis, evolutionary relationships between the different species of algae, as well as their unique properties and adaptations. Recently, there has been a renewed interest in the study of algal biochemistry and biology for their potential use in the development of renewable biofuels [reviewed in [12]]. This has promoted the study of varied biochemical processes in diverse algae, such as hydrogen metabolism, fermentation, lipid biosynthesis, photosynthesis and nutrient assimilation [13-20]. One of the most studied algae is *Chlamydomonas reinhardtii*. It has a sequenced genome that has been assembled into large scaffolds that are placed on to chromosomes [6]. For many years, *Chlamydomonas *has served as a reference organism for the study of photosynthesis, photoreceptors, chloroplast biology and diseases involving flagellar dysfunction [21-25]. Its transcriptome has recently been profiled by RNA-seq experiments under various conditions of nutrient deprivation [[26,27], unpublished data (Castruita M., et al.)].

While *Chlamydomonas *has been extensively characterized experimentally, annotation of its genome is still approximate. Although KEGG categorizes some *C. reinhardtii *gene models into biological pathways, other databases - such as Reactome [28] - do not directly provide information for proteins of this green alga. Complicating the analysis of *Chlamydomonas *genes is the fact that there are two assemblies of the genome in use (version 3 and version 4) and multiple sets of gene models have been developed that are catalogued under diverse identifiers: Joint Genome Institute (JGI) FM3.1 protein IDs for the version 3 assembly, and JGI version FM4 protein IDs and Augustus version 5 IDs for the version 4 assembly [11,29]. The differences between these assemblies are significant; for example, the version 3 assembly contains 1,557 continuous segments of sequence while the fourth version contains 88. Although the version 3 assembly is superseded by version 4, users presently access version 3 because of the richer user-based functional annotations. In addition, other sets of gene predictions have been generated using a variety of additional data, including ESTs and RNA-seq data, to more accurately delineate start and stop positions and improve upon existing gene models. One such gene prediction set is Augustus u10.2. As such, there are a variety of gene models between different assemblies being simultaneously used by researchers, presenting complications in genomics studies. To facilitate the analysis of *Chlamydomonas *genome-scale data, we developed the Algal Functional Annotation Tool, which provides a comprehensive analysis suite for functionally interpreting *C. reinhardtii *genes across all available protein identifiers. This web-based tool provides an integrative data-mining environment that assigns pathway, ontology, and protein family terms to proteins of *C. reinhardtii *and enables term enrichment analysis for lists of genes. Expression data for several experimental conditions are also integrated into the tool, allowing the determination of overrepresented differentially expressed conditions. Additionally, a gene similarity search tool allows for genes with similar expression patterns to be identified based on expression levels across these conditions.

## Construction and Content

### Integration of Multiple Annotation Databases

The Algal Functional Annotation Tool integrates annotation data from the biological knowledge bases listed in Table 1. Publically available flat files containing annotation data were downloaded and parsed for each individual resource. *Chlamydomonas reinhardtii *proteins were assigned KEGG pathway annotations by means of sequence similarity to proteins within the KEGG genes database [1]. MetaCyc [2], Reactome [28], and Panther [30] pathway annotations were assigned to *C. reinhardtii *proteins by sequence similarity to subsets of UniProt IDs annotated in each corresponding database. In all cases, sequence similarity was determined by BLAST. BLAST results were filtered to contain only best hits with an E-value < 1e-05.

**Table 1:** List of annotation resources integrated into the Algal Functional Annotation Tool

Resource	URL	Reference
KEGG	http://www.genome.jp/kegg/	[1]
MetaCyc	http://www.metacyc.org/	[2]
Pfam	http://pfam.sanger.ac.uk	[3]
Reactome	http://www.reactome.org/	[28]
Panther	http://www.pantherdb.org/pathway	[30]
Gene Ontology	http://www.geneontology.org/	[31]
InterPro	http://www.ebi.ac.uk/interpro	[32]
MapMan Ontology	http://mapman.gabipd.org/	[33]
KOG	http://www.ncbi.nlm.nih.gov/COG/grace/shokog.cgi	[35]

Primary databases used to functionally annotate gene models and integrated into the Algal Functional Annotation Tool.

Gene Ontology (GO) [31] terms were downloaded from the *Chlamydomonas reinhardtii *annotation provided by JGI. These GO terms were associated with their respective ancestors in the hierarchical ontology structure to include broader functional terms and provide a complete annotation set. Pfam domain annotations were assigned by direct search against protein domain signatures provided by Pfam. InterPro [32] and user-submitted manual annotations are based on those contained within JGI's annotation of the *C. reinhardtii *genome [11]. These methods were applied to four types of gene identifiers commonly used for *C. reinhardtii *proteins: JGI protein identifiers (versions 3 and 4) and Augustus gene models (versions 5 and 10.2). In total, over 12,600 unique functional annotation terms were assigned to 65,494 *C. reinhardtii *gene models spanning four different gene identifier types by these methods (Table 2). These assigned annotations may be explored for single genes using a built-in keyword search tool as well as an integrated annotation lookup tool which displays all annotations for a particular identifier.

**Table 2:** Number of gene identifiers associated with annotation databases

Identifier Type	Total Gene IDs	KEGG	Reactome	Panther	Gene Ontology	MapMan	KOG	Pfam	InterPro
JGI v3.0	14598	5348	2740	1147	6563	5214	9139	7166	7532
JGI v4.0	16706	4232	1949	1085	7568	3171	9973	7305	8151
Augustus v5.0	16888	4686	2983	1673	4334	3160	5123	8202	5202
Augustus u10.2	17302	4583	3326	1913	6956	3892	8977	8691	7464

Number of *Chlamydomonas reinhardtii *identifiers with at least one functional annotation for each primary database, shown per identifier type.

### Assignment of Annotation from *Arabidopsis thaliana*

To extend the terms associated with *C. reinhartdii *genes, functional terms were inferred by homology to the annotation set of the plant *Arabidopsis thaliana *(thale cress). Identification of orthologous proteins was based on sequence similarity and subsequent filtering of the results by retaining only mutual best hits between the two sets of protein sequences. The corresponding *Arabidopsis thaliana *annotation was used to supplement GO terms and was similarly expanded to contain term ancestry. The *A. thaliana *annotations of the MapMan Ontology [33] and MetaCyc Pathway database [2] were also used to provide more complete annotation coverage of the *C. reinhardii *genome.

### Functional Term Enrichment Testing

The hypergeometric distribution is commonly used to determine the significance of functional term enrichment within a list of genes. In this test, the occurrence of a functional term within a gene list is compared to the background level of occurrence across all genes in the genome to determine the degree of enrichment. A p-value based on this test can be calculated from four parameters: (1) the number of genes within the list, (2) the frequency of a term within the gene list, (3) the total number of genes within the genome, and (4) the frequency of a term across all genes in the genome. This test effectively distinguishes truly overrepresented terms from those occurring at a high frequency across all genes in the genome and therefore within the gene list as well. The cumulative hypergeometric test assigns a p-value to each functional term associated with genes within a given list, and all functional terms are ranked by ascending p-value (i.e. by descending levels of enrichment). Huang et al. reviews the use of the hypergeometric test for functional term enrichment [34]. The Algal Functional Annotation Tool computes hypergeometric p-values using a Perl wrapper for the GNU Scientific Library cumulative hypergeometric function written in C to provide a quick and accurate implementation of this statistical test.

### Dynamic Visualization of KEGG Pathway Maps

Individual pathway maps from KEGG provide information on protein localization within the cell, compartmentalization into different cellular components, or of reactions within a larger metabolic process. Visualization of proteins from gene lists onto pathway maps is useful for their interpretation. The Algal Functional Annotation Tool utilizes the publicly available KEGG application programming interface (API) for pathway highlighting. The information linking *C. reinhardtii *proteins to identifiers within the KEGG database is used to determine the subset of KEGG IDs within the supplied gene list associated with a particular pathway. The Algal Functional Annotation Tool also deduces which proteins within the pathway are located within the genome of *C. reinhardtii *but not found in the gene list and sends the corresponding identifiers to the KEGG API to be highlighted in a different background color. This API interface is implemented using the SOAP architecture for web applications.

### Integration of Expression Data

The expression levels of *C. reinhardtii *genes have been experimentally characterized under numerous conditions using high-throughput methods such as RNA-seq [[26,27], unpublished data (Castruita M., et al.)]. These expression data were compiled and analyzed to determine which genes are over- and under-expressed in each experimental condition. The expression data was preprocessed to normalize the counts for uniquely mappable reads in any experiment. Genes exhibiting greater than a two-fold change in expression compared to average expression across all conditions with a Poisson cumulative p-value of less than 0.05 were considered differentially expressed. Using this data, *C. reinhardtii *genes were associated with conditions in which they were over- and under-expressed.

The compiled expression data was also analyzed to find functionally related genes based on their expression levels across the different experimental conditions [[26,27], unpublished data (Castruita M., et al.)]. Genes demonstrating low variance of expression across all samples were not considered. This analysis was performed for three representations of the expression data: absolute counts, log counts, and log ratios of expression. By this method, *C. reinhardtii *genes are each associated with 100 genes with the most similar expression patterns to determine potentially functionally related genes.

### Gene Identifier Conversion

Due to the existence of several protein identifier types (FM3.1, FM4, Au5, Au10.2), different identifiers are associated with an individual protein within the *Chlamydomonas *genome. In order to extend annotations from one identifier type to another, matching protein identifiers are deduced by sequence similarity filtering for mutual best hits between identifiers using BLAST. Matching identifiers with 100% sequence coverage are kept, and the rest of the mutual best hits are filtered to include only those proteins with matches with at least 75% coverage. Potential ambiguities involving proteins similar to multiple other proteins are resolved by considering only the reciprocal best hit from the BLAST query in the opposite direction. The information derived by this analysis is used to convert gene identifiers between different types, which allows the Algal Annotation Tool to work with multiple protein identifier types.

### Web-Based Interface and Updates

The web interface of the Algal Functional Annotation Tool consists of a set of portals that give access to the different types of analyses available. Results are shown within expandable/collapsible HTML tables that display annotation information along with the statistical results of the analysis. When expanded, the results table shows which gene identifiers contain a specific annotation along with further information regarding matching gene identifiers and BLAST E-values. Updates to the Algal Functional Annotation Tool are semi-automated using a set of Perl scripts that parse and process updated flat files from the various integrated annotation databases at regular intervals. Currently, functional data from the primary annotation databases is set to be updated every 4 months.

## Utility and Discussion

### Comprehensive, Integrated Data-Mining Environment

**Figure 1:**
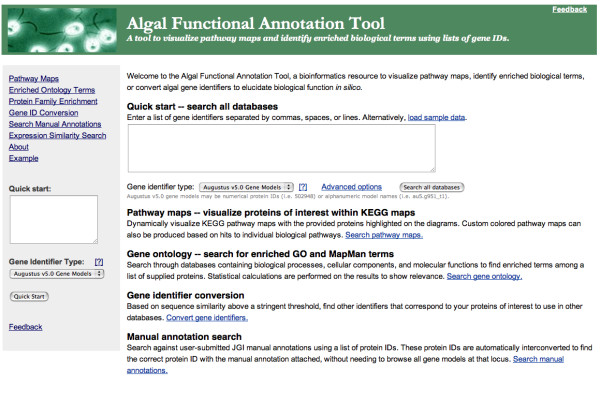
**Algal Functional Annotation Tool**. The front page of the Algal Functional Annotation Tool. A 'Quick Start' analysis is available to test for enrichment using the richest annotation databases included in the tool. Other features accessible from the sidebar include more specific enrichment tests (based on biological pathways, ontology terms, or protein families), a gene identifier conversion tool, a manual annotation search tool, and an expression similarity search tool.

The Algal Functional Annotation Tool is composed of three main components - functional term enrichment tests (which are separated by type), a batch gene identifier conversion tool, and a gene similarity search tool. A 'Quick Start' analysis is provided from the front page, featuring enrichment analysis using a sample set of databases containing the richest set of annotations (Figure 1). From any page, the sidebar provides access to the 'Quick Start' function of the tool.

**Figure 2:**
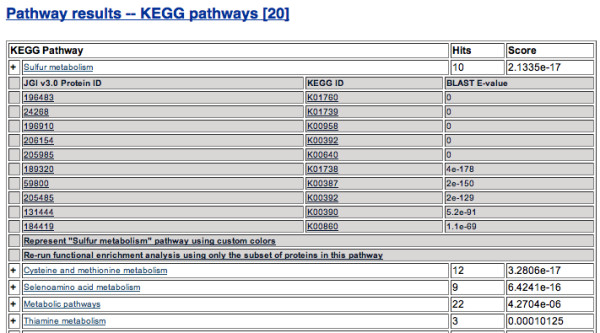
**Annotation Enrichment Results**. Annotation enrichment results, sorted by ascending hypergeometric p-values, are shown in expandible/collapsible HTML tables such as the one shown. When expanded, the genes within the user-submitted list containing the expanded annotation are shown alongside additional statistical information. All results are downloadable as tab-delimited text files.

Numerous other enrichment analyses - including enrichment using pathway, ontology, protein family, or differential expression data - are available within the Algal Functional Annotation Tool. Enrichment results are always sorted by hypergeometric p-value and whenever possible contain links to the primary database's entry for that annotation or to the protein page of the gene identifier. The number of hits to a certain annotation term are also displayed alongside the p-value, and results may always be expanded to show additional details, such as the specific gene IDs within the list matching a certain annotation (Figure 2). These results are downloadable as tab-delimited text files which may then be further analyzed or used in conjunction with other databases.

**Figure 3:**
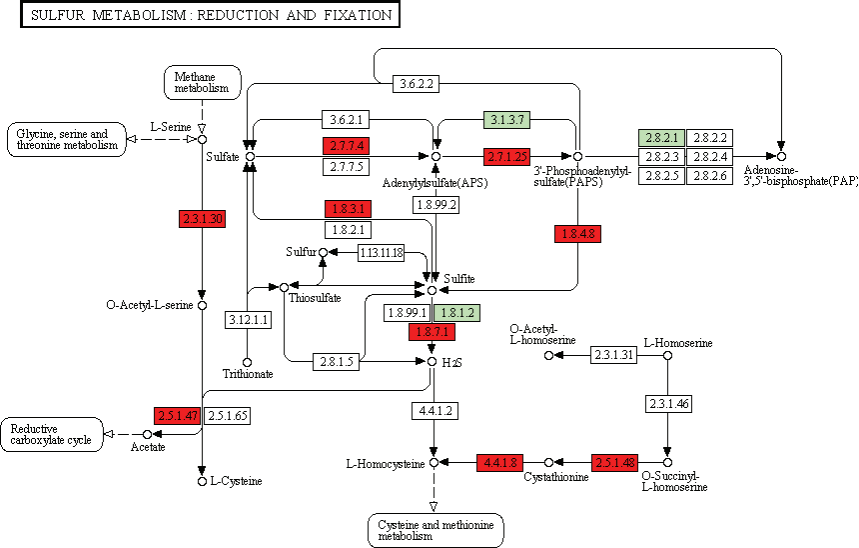
**Dynamic Visualization of Gene Lists onto KEGG Pathway Maps**. Dynamic KEGG pathway maps may be visualized to show the different proteins within a user-submitted gene list. Shown is the 'Sulfur Metabolism' dynamic pathway with the matching proteins submitted highlighted in red. In this example, the submitted gene list is drawn from literature characterizing *Chlamydomonas *under sulfur-deprived conditions [26].

Dynamic visualization of KEGG pathway maps may be accessed from the results table for KEGG pathway enrichment by clicking on any pathway name. The proteins in the list that are members of the particular biological pathway will appear in red, while those proteins existing in *Chlamyomonas reinhardtii *but not in the list appear in green (Figure 3). Alternatively, by expanding the pathway results and following the link at the bottom, the user may select a custom color scheme for visualizing the proteins on pathway maps. These custom color schemes may be designed on a gene-by-gene basis (choosing colors individually for genes) or in a group-by-group fashion (such as choosing a color for those proteins found within the organism but not in the gene list).

A list of genes may also be converted into a list of gene identifiers of another type. This feature allows easy transformation of gene IDs into corresponding models for use in other databases that may have additional annotation information. Additionally, the resulting list of gene identifiers may be used as a new starting point for enrichment analysis. Because of the different annotations associated with other gene identifier types (albeit of the same proteins), enrichment results using a converted set of gene IDs may yield new biological information.

**Figure 4:**
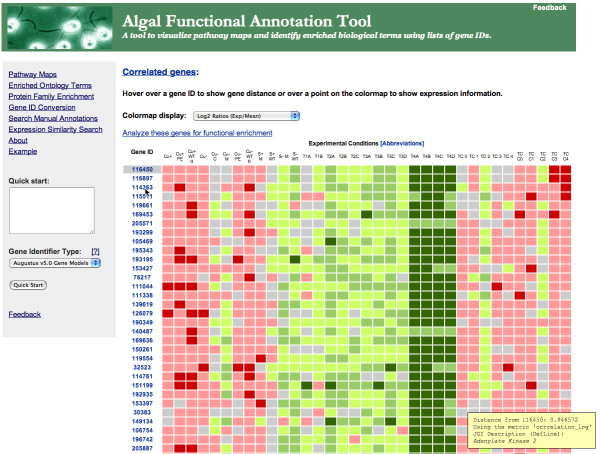
**Expression Similarity Search Tool Results**. An example of the results from the Gene Similarity Search Tool. Pairwise distances between resulting genes and the submitted gene are shown in the lower right corner when the mouse hovers over a gene of interest. Whenever applicable, a short description of the resulting gene is also shown when hovering over a gene. Expression data is shown when hovering over a point of the colormap.

The gene similarity search tool, the third component of the Algal Functional Annotation Tool, accepts single genes and returns functionally related genes (based on gene expression across different experimental conditions) using user-specified distance metrics and thresholds. Presently, functionally related genes may be determined using correlation distance based on absolute counts, log counts, or log ratios of expression. The results page shows the original query gene at the top in gray and any resulting genes, sorted by similarity, are shown below the query gene (Figure 4). A colormap based on gene expression is generated for the different genes across the conditions, and this colormap may be changed to display absolute expression, log expression, or log ratios of expression. The distance between any gene and the original query gene is displayed by hovering the mouse over the gene identifier of interest. Quantitative expression data (e.g. absolute counts) are provided for each experiment by hovering over the colormap. Whenever a description of a gene is available, this is displayed when hovering over the gene identifier as well. Links to external databases (e.g. JGI, KEGG) providing more information about the genes are provided with the results.

### Ability to Re-Run Analysis for Subsets of Genes

Once a gene list is supplied and enrichment results have been returned, a subset of genes corresponding to those that contain a particular annotation may be isolated and re-run through the tool to be analyzed as a separate, smaller gene list. This allows users to select a particularly interesting group of functionally related genes and isolate them to see if they are also enriched for other functional terms. This also allows the user to prune large gene lists into more focused lists of functionally similar genes and removing some of the inherent noise associated with high-throughput experimental techniques and their resulting gene lists. This feature of the tool may be accessed by expanding the enrichment results of a particular annotation and selecting to re-run the analysis using only that subset of proteins. From this step, users may select which database types to query for enrichment (e.g. pathway, ontology, protein family).

### Expanded Annotation Coverage

The methods described to compensate for the incomplete annotation coverage of *Chlamydomonas reinhardtii *genes resulted in the addition of a vast number of unique annotations to the genome. While there is a strong overlap between pre-existing annotations and those assigned by inference, many new terms have also been added. The annotations derived by orthology, however, are not mixed with the annotations attained directly to decrease the possibility of false positive associations of functional terms that may distort the analysis, and to permit a comparison with the functional terms derived directly from the *Chlamydomonas *annotation.

### Example - Sulfur-Related Genes

Using a filtered list of *C. reinhardtii *genes derived from transcriptome sequencing of the green alga under sulfur-depleted conditions [26], the Algal Functional Annotation Tool found enrichment for annotations related to sulfur metabolism, cysteine and methionine metabolism, and sulfur compound biosynthesis. For each annotation, the results may be expanded to reveal the genes containing that particular annotation. Furthermore, there is significant overlap between terms directly assigned to *C. reinhardtii *proteins and those inferred from *A. thaliana *orthology. Visualization of the sulfur metabolism KEGG pathway shows that a majority of the enzymes involved in this biological process is in the sample list, and the reactions they catalyze may be seen on the pathway map. The results for any enrichment analysis may be downloaded as a tab-delimited text file. Taking a gene found to be associated with the KEGG pathway 'Sulfur metabolism' by this enrichment analysis (JGI v. 3 ID 206154) as a starting input into the gene similarity search tool, the genes corresponding to sulfate transporter, methionine synthase reductase, and cysteine dioxygenase were found within the top 15 results using the correlation metric between log counts.

### Future Directions

As with all tools that integrate data from multiple external sources, the power of analysis using the Algal Functional Annotation Tool is ultimately limited by the quality of the annotations within the primary databases. With the steady growth of knowledge in these annotation databases, the utility of the analyses provided is expected to increase in the future as more biological associations are assigned to genes. Additionally, as *Chlamydomonas reinhardtii *genes continue to be experimentally characterized, the assignment of manual annotations will also fill in the gaps left by automated annotation assignment and thus expand the annotation coverage throughout the genome, further improving the results generated by our portal. Lastly, the extensible nature of the Algal Functional Annotation Tool will allow us to add other algal organisms in the future using the same platform so that genomic data from other algal model organisms may be analyzed in a similar fashion as that currently available for *Chlamydomonas reinhardtii*.

## Conclusions

The Algal Functional Annotation Tool is intended as a comprehensive analysis tool to elucidate biological meaning from gene lists derived from high-throughput experimental techniques. Annotation sets from a number of biological databases have been pre-processed and assigned to gene identifiers of the green alga *Chlamydomonas reinhardtii*, and this annotation data may be explored in multiple ways, including the use of enrichment tests designed for large gene lists. Furthermore, the site enables the visualization of proteins within pathway maps. Using several methods, such as inferring annotations from orthologous proteins of other organisms, the initially sparse annotation coverage of *C. reinhardtii *is alleviated, allowing for a more effective functional term enrichment analysis. Other functions of the tool include a batch gene identifier conversion tool and a manual annotation search tool. Lastly, similar genes based on expression across several conditions may be explored using the gene similarity search tool.

## Availability and Requirements

Project name: Algal Functional Annotation Tool

• Public web service: http://pathways.mcdb.ucla.edu; Free and no registration.

• Programming language: Perl/CGI

• Database: MySQL

• Software License: GNU General Public License

## List of Abbreviations Used

API: Application Programming Interface; BLAST: Basic Local Alignment Search Tool; CGI: Common Gateway Interface; DAVID: Database for Annotation, Visualization, and Integrated Discovery; GO: Gene Ontology; KEGG: Kyoto Encyclopedia of Genes and Genomes; JGI: Joint Genome Institute; SOAP: Simple Object Access Protocol.

## Authors' contributions

MP conceived the analysis and main features of the tool. DL wrote and tested the code, constructed the annotation database, designed the user interface, and wrote the initial draft of the manuscript. SC provided the implementation of the hypergeometric distribution function. DC provided Pfam data and compiled the expression data. SM provided access to the expression data and tested the tool. All authors read, edited and approved the final manuscript.
